# Phased secondary small interfering RNAs in *Panaxnotoginseng*

**DOI:** 10.1186/s12864-017-4331-0

**Published:** 2018-01-19

**Authors:** Kun Chen, Li Liu, Xiaotuo Zhang, Yuanyuan Yuan, Shuchao Ren, Junqiang Guo, Qingyi Wang, Peiran Liao, Shipeng Li, Xiuming Cui, Yong-Fang Li, Yun Zheng

**Affiliations:** 1 Faculty of Life Science and Technology, Kunming University of Science and Technology, Kunming, 650500 China; 2 Yunnan Key Laboratory of Primate Biomedical Research, Institute of Primate Translational Medicine, Kunming University of Science and Technology, Kunming, 650500 China; 3 Faculty of Information Engineering and Automation, Kunming University of Science and Technology, Kunming, 650500 China; 4 College of Life Sciences, Henan Normal University, Xinxiang, 453007 China; 5 Key laboratory of Panax notoginseng resources sustainable development and utilization of state administration of traditional Chinese medicine, Kunming, 650500 China; 6 Provincial key laboratory of Panax notoginseng of Yunnan, Kunming, 650500 China

**Keywords:** PHAS, Phased small interfering RNA (phasiRNA), *Panax notoginseng*, High-throughput sequencing, Bioinformatics

## Abstract

**Background:**

Recent results demonstrated that either non-coding or coding genes generate phased secondary small interfering RNAs (phasiRNAs) guided by specific miRNAs. Till now, there is no studies for phasiRNAs in *Panax notoginseng* (Burk.) F.H. Chen (*P. notoginseng*), an important traditional Chinese herbal medicinal plant species.

**Methods:**

Here we performed a genome-wide discovery of phasiRNAs and its host PHAS loci in *P. notoginseng* by analyzing small RNA sequencing profiles. Degradome sequencing profile was used to identify the trigger miRNAs of these phasiRNAs and potential targets of phasiRNAs. We also used RLM 5’-RACE to validate some of the identified phasiRNA targets.

**Results:**

After analyzing 24 small RNA sequencing profiles of *P. notoginseng*, 204 and 90 PHAS loci that encoded 21 and 24 nucleotide (nt) phasiRNAs, respectively, were identified. Furthermore, we found that phasiRNAs produced from some pentatricopeptide repeat-contain (PPR) genes target another layer of PPR genes as validated by both the degradome sequencing profile and RLM 5’-RACE analysis. We also found that miR171 with 21 nt triggers the generations of 21 nt phasiRNAs from its conserved targets.

**Conclusions:**

We validated that some phasiRNAs generated from PPRs and TASL genes are functional by targeting other PPRs in *trans*. These results provide the first set of PHAS loci and phasiRNAs in *P. notoginseng*, and enhance our understanding of PHAS in plants.

**Electronic supplementary material:**

The online version of this article (doi:10.1186/s12864-017-4331-0) contains supplementary material, which is available to authorized users.

## Background

Plant small RNAs, with 21 to 24 nucleotides (nt), play crucial roles in a variety of biological processes, including development, stress responses, defense and epigenetic modifications [1, 2]. Based on the origin and biogenesis, small RNA in plants can be divided into two main categories: microRNA (miRNA) and small interfering RNA (siRNA); siRNA can be further divided into three classes: heterochromatic siRNA (hc-siRNAs), natural antisense transcript siRNA (NAT-siRNA) and phased, secondary siRNA (phasiRNA) [3, 4]. The common feature of these small RNA is that they are generated by different DICER-LIKE (DCL) member from their long precursors, and then are incorporated into an effector complex to guide target recognition and subsequent silencing events in a sequence-specific manner [4, 5].

MicroRNAs are derived from their precursors, which are non-coding RNAs and can form hairpin-like secondary structures. The miRNA precursors are generated by RNA polymerase II (Pol II); RNase III enzyme DCL1 is responsible for the biogenesis of the mature miRNAs via processing of miRNA precursors. Most miRNAs negatively regulate their target genes through homolog based mRNA cleavage or translation inhibition at post-transcriptional level [2, 6], however some miRNAs may activate their targets through different mechanisms [7–9]. Small interfering RNAs (siRNAs) are characterized by their biogenesis depending on different RNA-dependent RNA polymerase (RDR) member. hc-siRNAs are 24 nucleotides in length, generated by DCL3 activity [10]. hc-siRNAs play a crucial roles in directing DNA methylation and histone modification in an AGO4-dependent pathway, and regulate target gene expression in transcription level [11, 12]. NAT-siRNAs derives from dsRNA precursors formed by bidirectional transcription of two partially overlapping genes [13–15]. PhasiRNA is a kind of secondary siRNA. Phase simple means these siRNAs are generated precisely in phased pattern initiated at a specific nucleotide. Its biogenesis requires an initiative cleavage on the phasiRNA precursor transcript (PHAS) by a specific miRNA in either “one-hit" or “two-hit" manner, then one of the cleaved product is made double stranded by RDR6 and then catalyzed by DCL4 into 21 nt siRNA in a phased pattern [16–20]. Some of the phasiRNAs may further target their parental genes in *cis* or other genes in *trans* [21].

PhasiRNAs can be generated from either long noncoding RNAs or coding genes. Arabidopsis miR173 (*TAS1* and *TAS2*), miR390 (*TAS3*), and miR828 (*TAS4*) can function as guides on non-coding primary transcripts to initiate tasiRNA1-2, tasiRNA3 and tasiRNA4 processing, respectively. These tasiRNAs can further target pentatricopeptide repeat (PPR) family member, auxin response factors (ARFs) and MYB transcription factor in *trans* manner [17, 19, 22, 23]. Among them, tasiRNA3 is much conversed in land plants [16, 17]. Recently, 21- and 24-nt phasiRNAs derived from long noncoding RNAs have been reported in rice and maize male reproductive organs, they are trigged by miR2118 and miR2275, and cleaved by DCL4 and DCL5 (also known as DCL3b), respectively [20, 24]. A noncoding PHAS locus, triggered by miR4392, was found accumulating preferentially in soybean anthers [25].

PhasiRNAs, triggered by miRNAs, are also generated from protein-coding loci in many plants, and first found in Arabidopsis [26]. It is worthy to point out that a significant number of PPR, nucleotide binding site-leucine-rich repeat (NB-LRR) and MYB family members are PHAS loci [21, 26–29]. PPR family is one of the largest families in Arabidopsis, containing at least 448 PPR-related genes. Many PPR-P clade transcripts have been identified as PHAS loci triggered by miR161.1, miR161.2, miR400 and tasiRNAs produced by miR173-TAS1/2 [3, 26]. PPR PHAS loci have been found in 9 eudicots, and triggered by miR7122, miR1509 and fve-PPRtri1/2 [3]. MYB transcription factor are target by miR159/miR828/miR858 in apple, peach and lotus, MYB-derived phasiRNAs could target a variety of genes with different function [27, 29, 30]. NB-LRR is the largest family targeted by small RNA. Arikit et al. [25] found 500 PHAS loci in soybean, amazingly, 208 loci are NB-LRR genes; Zhai et al. [21] found 114 PHAS loci in Medicago, among them, 79 loci are NB-LRR PHAS loci and 74 NB-LRR loci are targeted by miR1507, miR2109 and miR2118. NB-LRR PHAS loci are found in many plant species, indicating a conserved role of phasiRNA in regulation of NB-LRR [29, 31]. NB-LRR can be targeted by both miRNAs and phasiRNAs [21]. Besides these three gene families, AP2-like gene was found as PHAS loci triggered by miR156 and miR172 in Arabidopsis, DCL2 and Suppressor of Gene Silencing 3 (SGS3) were designed as PHAS loci trigged by miR1515 and miR2118, respectively, in Medicago and soybean [21].

*TAS3* derived tasiARFs are the only phasiRNAs that have been validated to target ARF genes in *trans* [16, 17, 28]. The functions of most phasiRNAs are still largely unknown [3].

To investigate phasiRNAs in *Panax notoginseng*, a precious traditional Chinese herb, we analyzed 24 small RNA profiles of *P. notoginseng*, and identified 204 PHAS loci generating 21 nt phasiRNAs, and 90 PHAS loci generating 24 nt phasiRNAs. Via integrating miRNAs and parallel analysis of RNA end (PARE) data, we found that some PPR genes generate phasiRNAs, moreover, we found these phasiRNAs could target other PPR loci in *trans* manner. Using RLM 5’-RACE technique, we further validated the cleavage on PPR transcripts induced by phasiRNAs. We present direct evidence that phasiRNAs originated from PPR genes are functional to regulate other PPR genes in *trans*. Our results also suggest that miR171, with 21 nt, could trigger phasiRNA generation in wide type plants, although existing study found that miR171 with additional nucleotide at 3’ end could trigger phasiRNAs in a mutant.

## Methods

### Materials and small RNA sequencing profiles

We sequenced 7 small RNA sequencing profiles of *P. notoginseng* grown in Wenshan County, Yunnan, China (Additional file 1: Table S1). Two of these 7 samples was mixed RNA sample of the leaf and root. Other 5 samples were RNA samples extracted from roots of 5 different plants. The tissue samples collected were stored in liquid nitrogen immediately. The samples were kept at -80 °C until RNAs were extracted. Total RNAs were extracted from samples using the TRIzol reagent (Invitrogen, Thermo Fisher Scientific Inc., USA) according to the manufacturer’s protocol. The integrities of the RNAs were checked using an ultraviolet spectrophotometer (Hoefer, MA, USA), based on the ratio of the optical density at 260 nm to that at 280 nm (OD260/280) and were also assessed by electrophoresis in a denaturing formaldehyde agarose gel, based on visual comparison with the strength of 18S and 28S ribosomal RNAs. The RNA samples with OD260/280 between 1.8 and 2.0 were checked for the total quantities. Only samples with at least 20 *μ*g were chosen for preparation of sRNA sequencing libraries. The small RNAs of the samples were isolated from total RNAs and were sequenced using Illumina HiSeq 2000 sequencer. The 7 obtained small RNA sequencing profiles were deposited into the NCBI GEO database and are accessible with series ID GSE98118.

Because each plant sample may only express some parts of the whole gene set, it is very helpful to identify PHAS loci as completely as possible by analyzing sRNA profiles from multiple samples. Therefore, the 17 small RNA sequencing profiles downloaded from the NCBI SRA database with accession ID SRP082250 and the 7 profiles obtained in this study were combined to get unique sRNA sequences and normalized frequencies in each of the profiles.

### Computational steps for identifying PHAS loci

We aligned the unique sequences in the small RNA libraries to the cDNA sequences of *P. notoginseng* (Zheng and Cui, unpublished) with SOAP2 [32]. Then, we examined the distribution of unique 21 and 24 nt sRNAs on cDNA sequences with a window of 210 nt or 240 nt (ten 21 nt or 24 nt) respectively. The positions of sRNAs on the anti-sense strand were consider in the same phase as sRNAs on the sense strand with a two-nucleotide positive offset because sRNA duplex have two-nucleotide over-hang at the 3’-end [21, 26, 28, 33]. Then, the *P*-values of windows under consideration were calculated with a Hypergeometric distribution as proposed previously [33],

1 $$ P(X=k) = \sum\limits_{X=k}^{m}\frac{{20m \choose n-k}{m \choose k}}{{21m \choose n}},  $$

where *k* was the number of phased unique 21 nt (or 24 nt) sRNAs in the window, *n* was the total number of unique 21 nt (or 24 nt) sRNAs in the window, and *m* was the number of phases. In this study, *m* was set to 10.

We set the window sizes of PHAS loci according to the sizes of reported PHAS loci and the distances between the complementary sites of miRNA triggers to the phasiRNAs in other species. As reported previously [16, 17, 29], the functional tasiRNAs derived from TAS3 are at the seventh or eighth 21 nt segments from the 3’ complementary site of miR390. TAS4 derived functional and conserved tasiRNA, TAS4-siR81(-), is also very close the miR828 complementary site [23, 29]. For other PHAS loci and TAS1/2 in Arabidopsis, it has not been reported that these PHAS loci encode conserved phasiRNAs. But the phasiRNAs from these PHAS and TAS1/2 were very close to complementary sites of their miRNA triggers [17, 21]. Therefore, we set the window sizes of PHAS loci as ten 21 or 24 nt segments.

We calculated a phase score for each position of cDNA sequences using the method in [34]. For a window with at least three phased unique sRNAs, i.e., when *k*≥3,

2 $$ PhaseScore = \ln \left(1 + 10 \times \frac{\sum_{i = 1}^{m}P_{i}}{1+\sum_{i=1}^{m}U_{i}}\right)^{k - 2},  $$

where *m* was the number of phases in the window, *k* was the number of unique phased siRNAs in the window, *U*_*i*_ was the number of non-phased reads at the *i*th phase from the position, and *P*_*i*_ was the number of phased reads at the *i*th phase from the position. *m* was 10 in this study.

If a window has a *P*-value less than 0.05, it was extended 100bp at both 5’- and 3’-ends. Then we merged the overlapped windows. We next calculated the false discovery rates with the *P*-values of the merged windows with the method in [35]. We selected the merged windows with a maximal phase scores of 5 or more and multiple test corrected *P*-values less than 0.05 as putative PHAS loci. We named the predicted PHAS loci with its transcript ID and a increasing serial number. We reported the phased siRNAs of the predicted PHAS loci as phasiRNAs. We named the phasiRNAs of a PHAS loci by adding siR and a serial number to the name of the PHAS loci.

### Degradome sequencing

The degradome of a *P. notoginseng* root sample in our previous study was retrieved from NCBI SRA database using the accession number SRP087606. Briefly, the root of a *P. notoginseng* plants grown in a shading greenhouse at Kunming University of Science and Technology (24° 51 ^′^ 0 ^′′^ N, 102° 52 ^′^ 2 ^′′^ E, altitude 1835 m), Kunming, Yunnan, China, was frozen in liquid nitrogen immediately after harvesting. During the experiment, the daily average temperature was 25 °C, the daily maximum difference in temperature was 10 °C and humidity was 60-80%. The total RNA from the root of a *P. notoginseng* plant was extracted using the TRIzol reagent (Invitrogen, Thermo Fisher Scientific Inc., USA) according to the manufacturer’s protocol. The integrity of the RNA was checked with an ultraviolet spectrophotometry (Hoefer, MA, USA) and 2100 BioAnalyzer (Agilent Technologies, Santa Clara, CA, USA). The degradome of polyadenylated transcripts was sequenced using Illumina HiSeq 4000 sequencer. The obtained degradome sequencing profile was filtered to remove low quality reads that have low scored nucleotides (< 20). Then, the 3’ adapters in the remaining reads were removed.

### Identifying miRNA complementary sites on PHAS and phasiRNA targets

The degradome sequencing profile were downloaded from the NCBI SRA database with the accession number SRP087606. The raw degradome sequencing profile was filtered to remove low quality reads that have low scored nucleotides (< 20). Then, the 3’ adapters in the remaining reads were removed. The unique sequences were obtained and the frequencies of the unique sequences were calculated. The SeqTar algorithm [36] was used to predict miRNA complementary sites on the original transcripts of PHAS loci. For conserved miRNAs, the targets that have at least one valid read, i.e., read started at the 9th to 11th positions of a miRNA binding site (as defined in [36]), or targets that have less than 4 mismatches were used for further analysis.

We identified the targets of phasiRNAs with the SeqTar algorithm [36]. Only targets that have at least one valid read and less than 4 mismatches were used for further analysis.

### RLM 5’-RACE validations of some phasiRNA targets

Modified 5’-RACE assay was performed using the GeneRacer Kit (Invitrogen) to validate the predicted targets. Briefly, 5 *μ*g total RNA was ligated with GeneRacer ^*TM*^ RNA Oligo (5’-CGACUGGAGCACGAGGACACUGACAUGGACUGAAGGAGUAGAAA-3’) and reverse transcription was performed using SuperScript ^*TM*^ III Reverse Transcriptase and oligo dT(18) primer. The resulting cDNA was used as template for PCR amplification with GeneRacer 5’ primer (5’-CGACTGGAGCACGAGGACACTGA-3’) and a gene-specific reverse primer. A second nested PCR was performed using GeneRacer 5’ nested primer (5’-GGACACTGACATGGACTGAAGGAGTA-3’) and a gene specific nested primer. The amplified products were run on a 2% agarose gel, bands with expected size were purified using QIAquick^®^ Gel Extraction Kit (QIAGEN) and ligated onto T Vector pMD 19 (Simple)(Takara), following transformation and clone PCR, plasmids were isolated and subjected to Sanger sequencing. Gene specific primers used are listed in Additional file 1: Table S2.

## Results and discussion

### Samples and small RNA profiles of *P. notoginseng*

We collected 5 root samples and 2 whole plant samples of *P. notoginseng* grown in Wenshan County, Yunnan, China. The small RNAs of the samples were isolated from total RNAs and were sequenced using Illumina HiSeq 2000 sequencer (see Methods). The obtained sRNA profiles were combined with 17 root sRNA profiles from NCBI SRA database obtained with accession number SRP082250 to get unique sRNA sequences and their frequencies in each of the profiles.

### Twenty-one nt PHAS loci in *P. notoginseng*

The combined sRNA profiles of 23 samples were aligned to a self-assembled transcriptome database of *P. notoginseng* using SOAP2 [32]. Then, we used a self-written program to predict PHAS loci and phasiRNAs from the alignment result of SOAP2 based on methods proposed previously [33, 34] (see Materials and methods for details). Then we merged overlapped PHAS loci. As listed in Additional file 1: Table S3, we totally identified 204 PHAS loci encoding 21 nt phasiRNAs with a combined criterion of a phase score ≥ 5 and a multiple-test corrected *P*<0.05. These PHAS loci produced at least 2505 twenty-one nt phasiRNAs that were sequenced in the sRNA profiles used in the study (Additional file 1: Table S4).

**Fig. 1:**
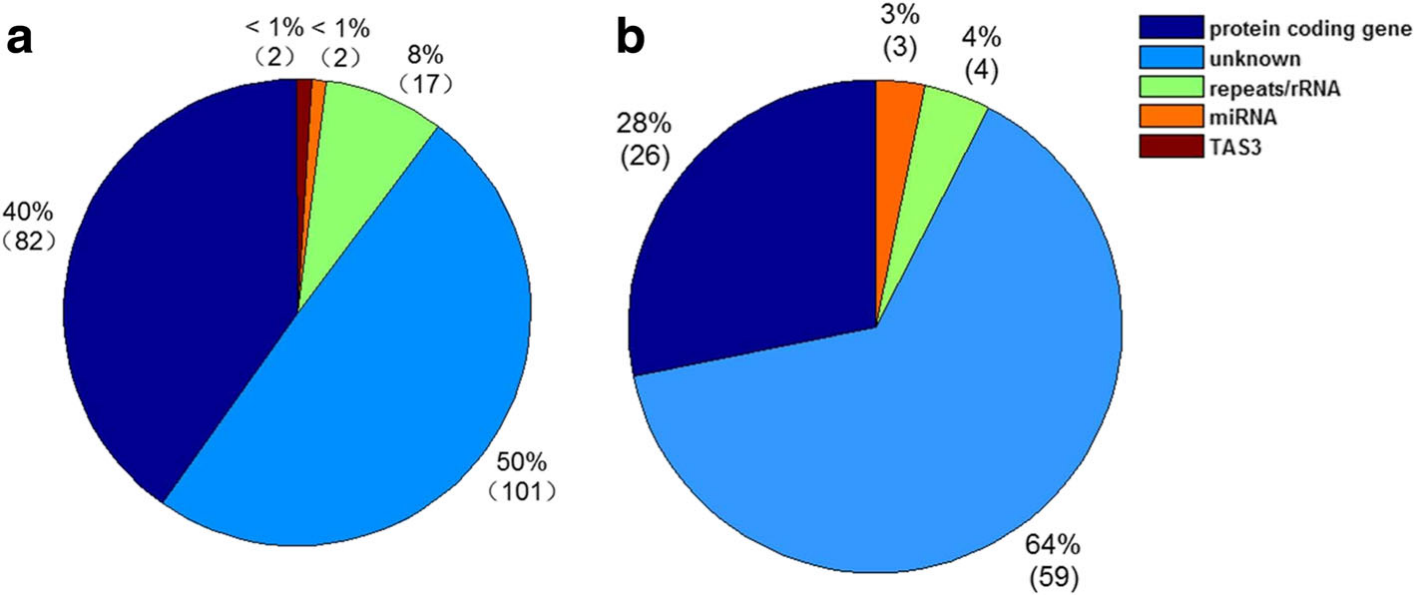
The types of molecules of predicted PHAS loci. **a** The types of molecules of PHAS loci generating 21 nt phasiRNAs. **b** The types of molecules of PHAS loci of PHAS loci generating 24 nt phasiRNAs

The predicted PHAS were annotated by aligning their sequences to the NCBI Nucleotide Collection (nr/nt) database and the TIGR Plant Repeat database (details are given in Additional file 1: Table S3). 50% of PHAS loci encoding 21 nt phasiRNAs are unknown genes (see Fig. 1a). The second largest type of PHAS loci encoding 21 nt phasiRNAs is protein coding genes, with 40% or 82 loci. Seventeen PHAS loci encoding 21 nt phasiRNAs were originated from rRNA or repeats. Two 21 nt PHAS loci are TAS3 genes and two PHAS loci encoding 21 nt phasiRNAs could be mapped to MIRNAs.

**Fig. 2:**
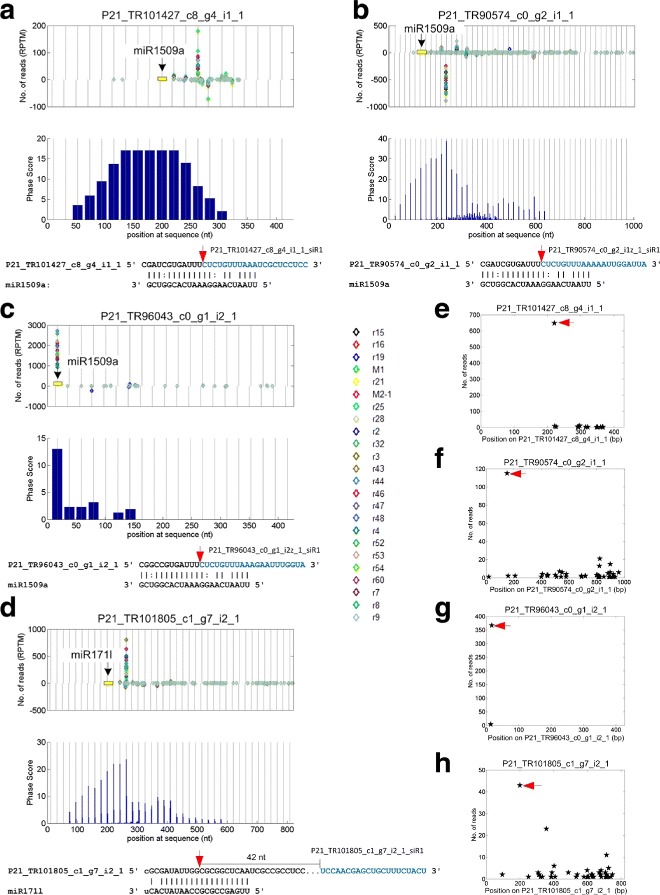
The distributions of sRNA and degradome reads, and phase scores of four 21 nt PHAS loci identified. From Part (**a**) to (**d**), the phased positions from the position with the highest phase scores of the PHAS loci were marked with the vertical gray lines. The miRNA complementary sites were marked as the yellow boxes in the read distribution panel. MiRNAs complement to the sense and anti-sense strand of the predicted PHAS loci were pointed by miRNAs from above and under zero read line, respectively. The predicted miRNA binding sites were illustrated under the panels of phase scores. **a** P21_TR101427_c8_g4_i1_1 that is targeted by miR1509a. **b** P21_TR90574_c0_g2_i1_1 that is targeted by miR1509a. **c** P21_TR96043_c0_g1_i2_1 that is targeted by miR1509a. **d** P21_TR101805_c1_g7_i2_1 that is targeted by miR171l. **e** to **h** are the distributions of degradome reads for PHAS loci in Part (**a**) to (**d**), respectively. In Part (**e**) to (**h**), the arrows correspond to the positions pointed by the arrows of the same colors in the lower panels of Part (**a**) to (**d**), respectively

Because miRNAs are critical in processing pathways of phasiRNAs, we identified miRNA triggers for these PHAS loci on both strands by using the degradome sequencing profile and the SeqTar algorithm [36]. At least two of the 21 nt PHAS loci were NB-LRR genes, and were triggered by miR2118 (Additional file 1: Table S3). Two TAS3 loci were triggered by miR390 (Additional file 1: Table S3). miR1509a triggers 16 PHAS loci (Additional file 1: Table S3), of which most are unknown genes. Three of the miR1509 targeted PHAS loci are shown in Fig. 2a to c, where it is shown that miR1509a guides the generation of the phasiRNAs in these PHAS loci. Furthermore, from the degradome profile, we found that miR1509a induces the cleavage of these PHAS transcripts from the centers of its complementary sites (Fig. 2e to g). Four more miR1509 triggered PHAS loci are shown in Additional file 2: Figure S1a to S1d. The degradome profile also clearly shows that miR1509 induces strong cleavages on these transcripts to initiate the production of phasiRNAs (Additional file 2: Figure S1e to S1h).

miR171l, with 21 nt, triggers two PHAS loci from its conserved targets in the scarecrow-like transcription factor family (Additional file 1: Table S3). One of these two PHAS loci, P21_TR101805_c1_g7_i2_1, is shown in Fig. 2d. Together with the degradome profile (Fig. 2h), it is shown that miR171l triggers the generation of phasiRNAs from the center of its complementary site on P21_TR101805_c1_g7_i2_1.

More over, miR482 also triggers phasiRNAs by targeting an unknown gene and a hypothetical protein (Additional file 2: Figures S1i and S1j, respectively).

The miRNA triggers for generation of phasiRNAs are mainly 22 nt, except the miR161, miR400, miR172 and miR390 with 21 nt (Table 1). miR161 and miR400 were only reported in *Arabidopsis* [26]. A previous study reported that miR171 with an additional 3’-U triggers production of phasiRNAs in the *hen1* mutant, however, miR171 with 21 nt could not trigger phasiRNA production in the wide type plant [37]. But our results suggest that miR171, with 21 nt, could also trigger the generation of phasiRNAs by targeting one of its conserved targets (TR101805 |c1_g7_i2) from the scarecrow-like (SCL) transcription factor family in *P. notoginseng* (Fig. 2d). To exclude the possibility that miR171 may have longer isoforms, we carefully examined the reads in the small RNA profiles and found the miR171 have as many as 20 isoforms that target the P21_TR101805_c1_g7_i2_1 (Additional file 2: Figure S2). But only four are highly expressed (Additional file 2: Figure S2), among which miR171b2-3p and miR171l have no 22 nt reads in the sequencing libraries, miR171f has a 22 nt read with less than 3 copies in most libraries, and miR171a2-3p targets a site 3 nt downstream of the miR171l site shown in Fig. 2d. Thus, miR171 members with 21 nt are the triggers for production of phasiRNAs from P21_TR101805_c1_g7_i2_1.

**Table 1:** The miRNA triggers that are reported to target PHAS loci

miRNA	Length (nt)	PHAS Loci	Species	References
miR161.1/.2, miR400	21	PPR	*Arabidopsis*	[26]
miR171	21	SCL	*P. notoginseng*	[37] and this study
miR172	21	AP2	*Medicago*	[21]
miR173	22	TAS1/2	*Arabidopsis*	[17]
miR390	21	TAS3	land plants	[16, 17] and this study
miR828	22	TAS4	*Arabidopsis*, lotus	[23, 29]
miR828	22	MYB	apple, peach, lotus	[27–30]
miR1507, miR1515	22	DCL2	*Medicago*, soybean	[21]
miR2118, miR482, miR1507,	22	NB-LRR	legume, cotton, lotus,	[20, 21, 29, 31, 39, 40]
miR2109			tomato, *P. notoginseng*	and this study
miR2118, miR2275	22	lncRNAs	rice, maize	[3, 20, 24]
miR5754	22	Protein kinase	*Medicago*	[21]
miR7122, miR1509, miR173	22	PPR	eudicots	[21, 26, 28] and this study

### Twenty-four nt PHAS loci in *P. notoginseng*

**Fig. 3:**
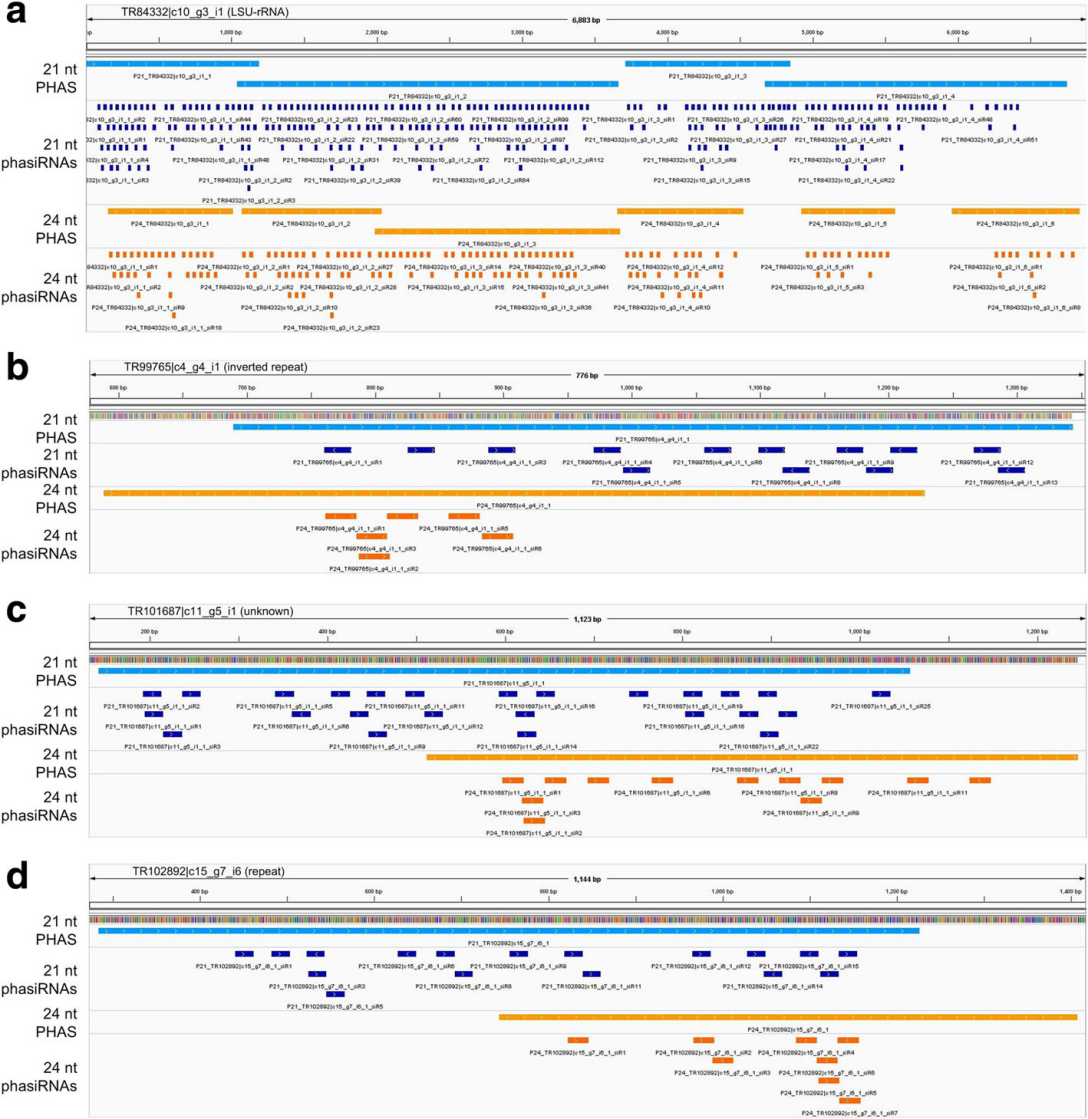
Four identified loci that produce both 21 and 24 nt phasiRNAs in *P. notoginseng*. The 21 nt PHAS/phasiRNAs and 24 nt PHAS/phasiRNAs are shown in blue and orange lanes, respectively. **a** TR84332 |c10_g3_i1, a Large Subunit rRNA (LSU-rRNA). **b** TR99765 |c4_g4_i1, an inverted repeat. **c** TR101687 |c11_g5_i1, an unknown gene. **d** TR102892 |c15_g7_i6, a repeat

In addition to 21 nt PHAS loci, we also found 90 PHAS loci (Additional file 1: Table S5) that generate over six hundred 24 nt phasiRNAs (Additional file 1: Table S6). Most of them, 66%, were unknown genes (Fig. 1b). 26 PHAS loci were from the protein coding genes (Fig. 1b). Three and four 24 nt PHAS loci are MIRNAs and rRNAs/repeats, respectively (Fig. 1b). Four 24 nt PHAS loci could also generate 21 nt phasiRNAs (Fig. 3). TR84332 |c10_g3_i1 in Fig. 3a is a Large Subunit rRNA (LSU-rRNA). In previous studies, we also found that rRNAs could produce both 21 nt and 24 nt phasiRNAs in lotus and pineapple [29, 38], suggesting that it is a conserved pathway to generate phasiRNAs, both 21 and 24 nt, from rRNAs. Two of the other three PHAS loci that produce 21 nt as well as 24 nt phasiRNAs were repeats (Fig. 3b and d). An unknown gene also produces both 21 and 24 nt phasiRNAs (Fig. 3c).

### PhasiRNA targets in *P. notoginseng*

We used the SeqTar algorithm [36] to identify targets of phasiRNAs by using a degradome profile of the *P. notoginseng* root. By keeping targets with at least 1 valid degradome read and have less than 4 mismatches, we found 5062 targets for 1699 twenty one nt phasiRNAs (Additional file 1: Table S7). In addition to 98 *cis*-targets, 4964 of these 5062 targets are *trans*-targets (Additional file 1: Table S7).

**Fig. 4:**
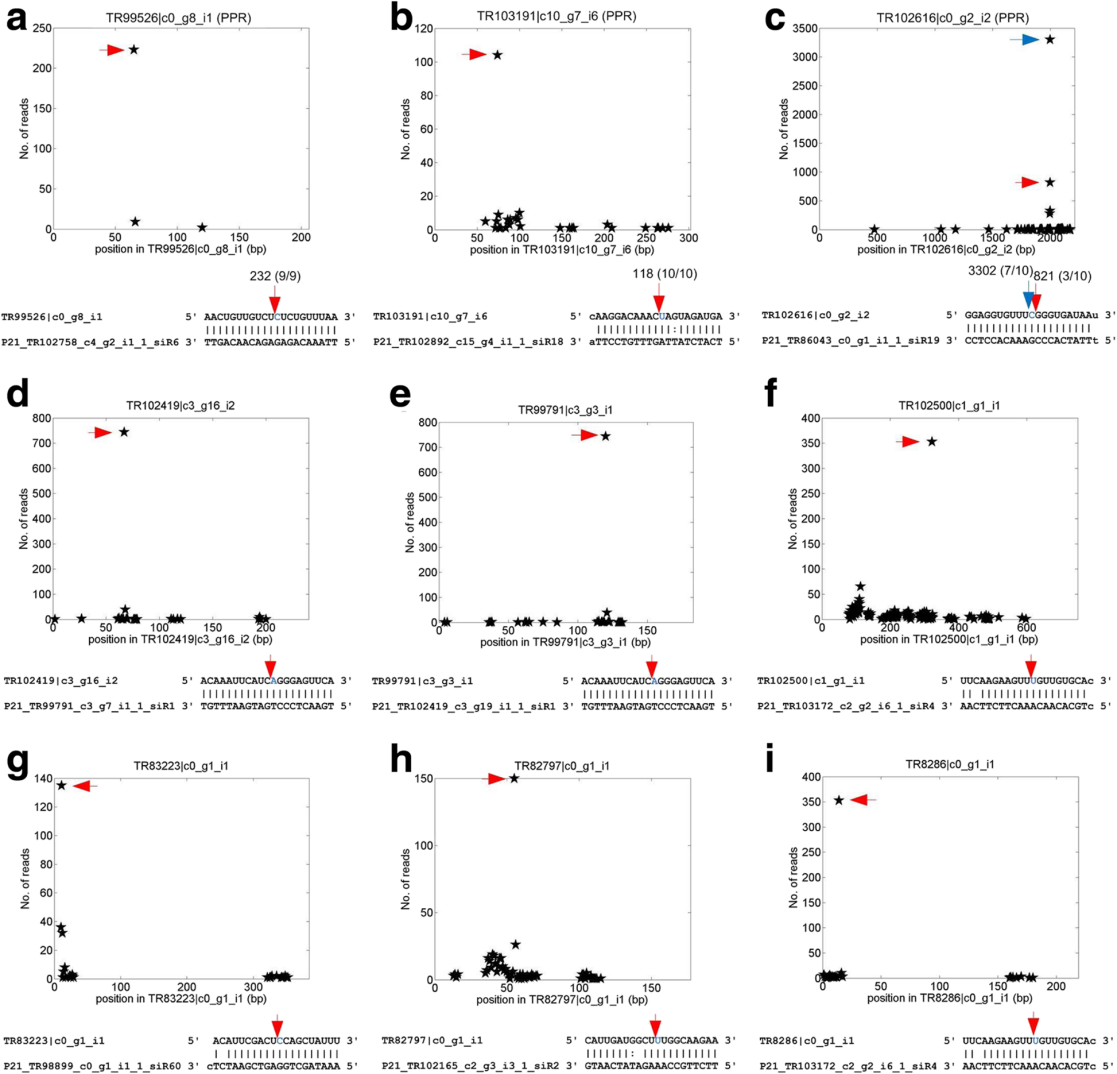
Identified targets of 21 nt phasiRNAs in *P. notoginseng*. The arrows in the upper panels correspond to the positions pointed by the arrows of the same colors in the lower panels. In Part (**a**) to (**c**), the numbers above the red arrows indicate the number of degradome reads from the position and the numbers in the parenthesis are the cleavage frequencies determined by the RLM 5’-RACE experiments. **a** TR99526 |c0_g8_i1, a putative PPR gene, targeted by P21_TR102758_c4_g2_i1_1_siR6. **b** TR103191 |c10_g7_i6, a putative PPR gene, P21_TR102892_c15_g4_i1_1_siR18. **c** TR102616 |c0_g2_i2, a putative PPR gene, P21_TR86043_c0_g1_i1_1_siR19. **d** TR102419 |c3_g16_i2, an unknown gene, targeted by P21_TR99791_c3_g7_i1_1_siR1. **e** TR8286 |c0_g1_i1, a putative ribosomal protein like gene, targeted by P21_TR103172_c2_g2_i6_1_siR4. **f** TR102500 |c1_g1_i1, a putative 60S ribosomal protein, targeted by P21_TR103172_c2_g2_i6_1_siR4. **g** TR83223 |c0_g1_i1, a putative heat shock protein like gene, targeted by P21_TR98899_c0_g1_i1_1_siR60. **h** TR82797 |c0_g1_i1, 60S putative ribosomal protein, targeted by P21_TR102165_c2_g3_i3_1_siR2. **i** TR102419 |c3_g16_i2, an unknown gene, targeted by P21_TR99791_c3_g7_i1_1_siR1

Some of the identified phasiRNA targets were shown in Fig. 4. As in Fig. 4a, a phasiRNA produced from a PPR loci, P21_TR102758_c4_g2_i1_1_siR6, targets another PPR gene (TR99526 |c0_g8_i1) (Additional file 1: Table S3 and Table S7). Two phasiRNAs, P21_TR102892_c15_g4_i1_1_siR18 and P21_TR86043_c0_g1_i1_1_siR19, produced from two unknown genes targets two different PPR genes, TR103191 |c10_g7_i6 and TR102616 |c0_g2_i2, respectively (Fig. 4b and c). Both the degradome profile and our RLM 5’-RACE experiments verified that the three phasiRNAs target their PPR genes in *P. notoginseng* (Fig. 4a to c). P21_TR102892_c15_g4_i1_1 is targeted by miR1509 (Additional file 1: Table S3). It has been reported previously that miR1509 target TASL genes to produce 21 nt phasiRNAs that subsequently target PPR genes [28]. We thus guess that TR102892_c15_g4_i1 might be a TASL gene.

TR102419 |c3_g16_i2 (an unknown gene), TR99791 |c3_g3_i1 (a PPR gene), TR102500 |c1_g1_i1 (a ribosomal protein), TR83223 |c0_g1_i1 (a heat shock protein), TR82797 |c0_g1_i1 (ubiquitin-60S ribosomal protein) and TR8286 |c0_g1_i1 (a ribosomal protein) are also identified as targets of different phasiRNAs (Fig. 4d to i). Interestingly, TR102419 |c3_g16_i2 and TR99791 |c3_g3_i1 produce the same phasiRNA and may form a mutual regulation loop or self-regulation loops through phasiRNAs (Fig. 4d and e).

In soybean and *Medicago truncatula*, miR1509 indirectly initiates the generation of phasiRNAs from PPR genes by one or two-layers of TASL-tasiRNA interactions [28]. In comparison, our results suggest that in addition to miR1509/TASL/PPR pathway, some PPR genes not triggered by miR1509 could also generate phasiRNAs to repress other PPRs in *P. notoginseng*. Presumably, these PPR PHAS loci are triggered by other unknown miRNAs or siRNAs.

One hundred twenty-nine targets of sixty one 24 nt phasiRNAs were also identified and filtered with the same criteria as those for the 21 nt phasiRNAs (Additional file 1: Table S8). Thirteen and 116 of these targets are *cis*- and *trans*-targets, respectively. But base on the degradome, only a few 24 nt phasiRNAs induce clear cleavage on the targets (Additional file 1: Table S8), suggesting these 24 nt phasiRNAs might function in other means than cleaving targets in *trans*, as noticed previously [20].

## Conclusion

**Fig. 5:**
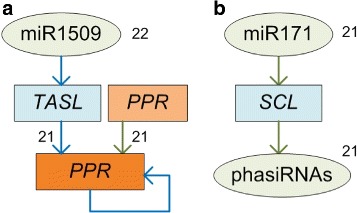
The new phasiRNA generation pathways identified in this study. Blue lines represent pathways reported in previous studies. Green lines represent pathways identified in this study. **a** The miR1509/TASL/PPR phasiRNA generation pathway. As reported previously [28], miR1509, with 22 nt, triggers the generation of phasiRNAs by targeting TASL transcripts, then phasiRNAs originated from the TASL transcripts target PPR genes. Here we found that some phasiRNAs derived from PPR genes could target other PPR genes in *trans*. **b** The miR171/SCL phasiRNA generation pathway

We found 204 and 90 PHAS loci that generate 21 and 24 nt phasiRNAs, respectively, in *P. notoginseng*. We found that some 21 nt phasiRNAs generated from PPR genes could target other PPRs in *trans* (see the green line in Fig. 5a), indicating that some of the 21 nt phasiRNAs are functional. Furthermore, although most miRNA triggers of PHAS are 22 nt (Table 1), we demonstrated that miR171, with 21 nt, triggers the generation of phasiRNAs from its conserved SCL targets in wide type *P. notoginseng* (Fig. 5b). This result is different from previously report that an edited miR171 with 22 nt could trigger phasiRNAs in a mutant plant [37]. These results provide the first set of PHAS loci and phasiRNAs in *P. notoginseng*, and enhance our understanding of PHAS in plants.

## Additional files

Additional file 1 Supplementary Tables. This is an MS Excel file with multiple sheets. This file includes 8 supplementary tables. (XLS 5603 kb)

Additional file 2 Supplementary Figures. This is a pdf file. This file includes 2 supplementary figures. (PDF 1148 kb)
