# Supplementary material for: Phased secondary small interfering RNAs in Panaxnotoginseng
Source: BMC Genomics. 2018 Jan 19;19(Suppl 1):41. doi: 10.1186/s12864-017-4331-0 (PMC5780745; doi:10.1186/s12864-017-4331-0)
Supplement: Supplementary file 2 — Supplementary Figures. This is a pdf file. This file includes 2 supplementary figures. (PDF 1148 kb) [file 12864_2017_4331_MOESM2_ESM.pdf]

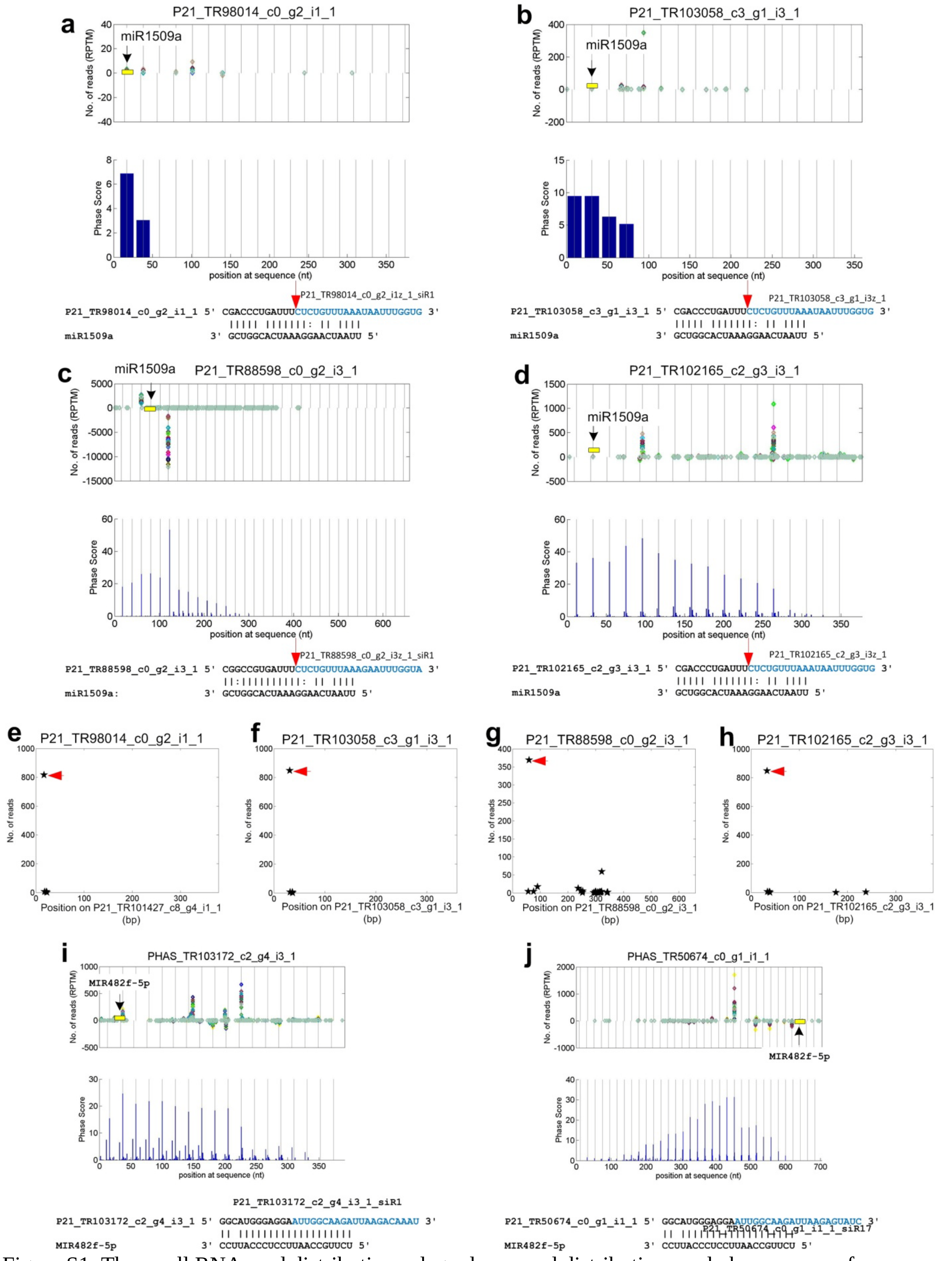

Figure S1. The small RNA read distributions, degradome read distributions and phase scores of some predicted 21 nt PHAS loci. From Part (a) to (d) and (i) to (j), the vertical gray lines with distances of 21 nt

are the phased positions from the position with highest phase scores of the PHAS loci. The yellow boxes in the read distribution panel represent the miRNA complementary sites. Sites pointed by miRNAs from above and under zero read line means miRNAs complement to the plus and minus strand of the predicted PHAS loci, respectively. The predicted miRNA complementary sites are shown below the phase score panel. The red arrows (a) P21_TR98014_c0_g2_i1_1 that is targeted by miR1509a. (b) P21_TR103058_c3_g1_i3_1 that is targeted by miR1509a. (c) P21_TR98014_c0_g2_i1_1 that is targeted by miR1509a. (d) P21_TR102165_c2_g3_i3_1 that is targeted by miR171l. (e) to (h) are the distribution of degradome reads for PHAS loci in Part (a) to (d), respectively. In Part (e) to (h), the arrows correspond to the positions pointed by the arrows of the same colors in the lower panels of Part (a) to (d), respectively. (i) P21_TR103172_c2_g4_i3_1 that is targeted by MIR482f-5p. (j) P21_TR50674_c0_g1_i1_1 that is targeted by MIR482f-5p.

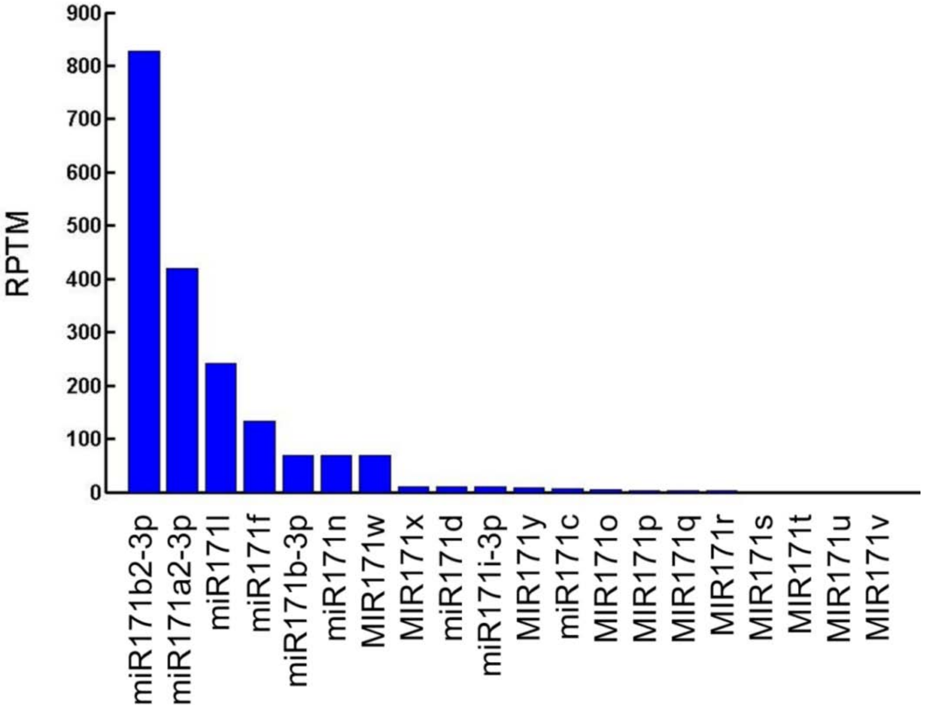

Figure S2. The summed normalized abundance (RPTM, Reads Per Ten Million sequencing tags) of miR171 isoforms.
